# The mitochondrial genome of Drino Sp. (Diptera, Tachinidae)

**DOI:** 10.1080/23802359.2018.1501318

**Published:** 2018-10-25

**Authors:** Peng Hou, Shuangmei Ding, Xin Li, Ding Yang, Chuntian Zhang, Qiang Wang

**Affiliations:** a College of Plant Protection, China Agricultural University, Beijing, China;; b College of Life Science, Shenyang Normal University, Shenyang, China;; c Shanghai Entry-Exit Inspection and Quarantine Bureau of China, Technical Center for Animal Plant and Food Inspection and Quarantine, Shanghai, China

**Keywords:** Calyptratae, Tachinidae, mitochondrial genome, phylogeny

## Abstract

The tachinid fly *Drino* sp. belongs to the subfamily Exoristinae of Tachinidae. We sequenced and annotated the mitogenome of *Drino* sp. which makes this species first representative of the tribe Eryciini (Tachinidae: Exoristinae) with nearly complete mitochondrial data. This mitogenome is 15437 bp in total, which consists of 22 transfer RNAs, 13 protein-coding genes, 2 ribosomal RNAs and non-coding control region. All genes have the conservational arrangement with other published species of Tachinidae. The nucleotide composition biases toward A and T, the overall A + T% was up to 80.4% of the entire mitogenome. Bayesian inference analysis strongly supported the monophyly of Tachinidae and Exoristinae. Our results also suggested that Exoristinae is the sister group to Phasiinae, and Dexiinae is the sister group to the clade of Phasiinae + Exoristinae.

Among all the species of flies, Tachinidae is one of the most diverse families with over 8,500 known species distributed worldwide. Tachinid flies play an important role in controlling the pests and balancing the ecosystem acting as enemy of pests (Stireman et al. 2006; O’Hara et al. 2009; O’Hara 2016; O’Hara and Cerretti 2016). Due to its various morphological features, entomologists face great challenge in identifying tachinid flies, the phylogenetic relationships in Tachinidae also draw the constant attention of the scholars (Meier et al. 2006; O’Hara 2013; Zhao et al. 2013).

Hence, we sequenced the mitochondrial genome of *Drino* sp., which could be the representative of subfamily Exoristinae for further research. Specimens of *Drino* sp. were collected in Mt. Wuling of Hebei Province by Kai Wang, and identified by Peng Hou and Prof. Chuntian Zhang. Specimens are deposited in the Entomological Museum of China Agricultural University (CAU).

The genomic DNA was extracted from the adult’s muscle tissues of the thorax using DNeasy DNA Extraction kit (TIANGEN, Beijing, China), and was sequenced under the next generation sequence technology. The mitochondrial genome of *Drino* sp. contains 22 transfer RNA genes, 13 protein-coding genes (PCGs), two ribosomal RNA genes and one non-coding control region, which were similar with related species reported before (Kang et al. 2016; Li et al. 2016, 2017; Wang et al. 2016a, 2016b, 2016c; Zhao et al. 2013).

The nucleotide composition of *Drino* sp. mitochondrial genome was 41.2% of A, 39.2% of T, 11.6% of C, and 8.0% of G. The A + T content was 80.4%, which was also at an average level among tachinid flies. The codon ATG was the most popular start codon shared with *ATP6*, *CO2*, *CO3*, *CYTB*, *ND4*, *ND4L*, and start codon ATT was shared with *ND2*, *ND3*, *ND5*, *ND6*. Particularly, the ATP8 begins with codon ATC, the *CO1* begins with codon TCG, and the ND1 begins with codon TTG. The conservative stop codon TAA was shared with *ATP6*, *ATP8*, *CO1*, *CO3*, *ND2*, *ND4L*, *ND6*, another common stop codon TAG was shared with *CYTB*, *ND1*, *ND3.* As for the other three genes, *CO2* and *ND5* were terminated with an incomplete stop codon T, while the gene *ND4* was ended with stop codon TA.

Phylogenetic analysis was performed on the basis of the dataset containing sequences of 13 PCGs from five Tachinidae and two Oestroidea species. The topology provided by Bayesian (BI) analysis based on the PCGs matrices was shown in Figure 1. According to the phylogenetic outcome, the outgroups *Lucilia sericata* (Calliphoridae) and *Sarcophaga crassipalpis* (Sarcophagidae) form a clade diverged from Tachinidae clades. Our results strongly supported the monophyly of Tachinidae as well as subfamily Exoristinae. It also indicated that subfamily Exoristinae is the sister group to subfamily Phasiinae, and subfamily Dexiinae is the sister group to the clade of Phasiinae + Exoristinae.

**Figure 1.**
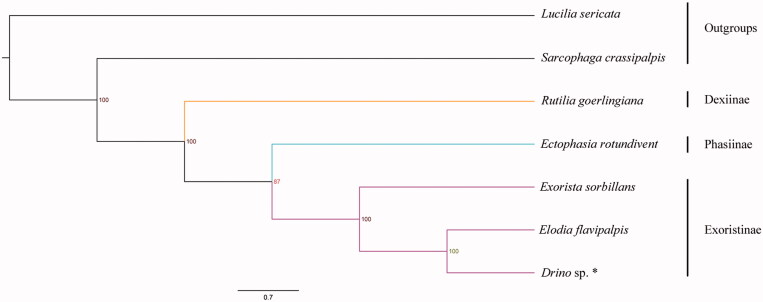
Bayesian phylogenetic tree of seven species which consist of five Tachinidae species and two outgroups. * indicates this study.
